# THE POSSIBILITY OF PREVENTION: THE PUBLIC HEALTH CENTER OF EXCELLENCE ON DEMENTIA RISK REDUCTION

**DOI:** 10.1093/geroni/igac059.273

**Published:** 2022-12-20

**Authors:** Matthew Baumgart

**Affiliations:** Alzheimer's Association, New York, New York, United States

## Abstract

While we may not be able to predict with certainty whether any particular individual can prevent dementia, we are now able to talk about dementia risk reduction at the population level. The Public Health Center of Excellence on Dementia Risk Reduction reviews and synthesizes the state of the science on modifiable risk factors for cognitive decline and dementia. The Center translates that science into public health action – identifying interventions and best practices to address those risk factors – and develops tools and resources for public health agencies. The Center disseminates this information and works with public health to undertake community-level actions to address risk factors. The Center also works with public health agencies to: (a) address the social determinants of health with respect to dementia risk; (b) build capacity to enable smaller agencies to engage in risk reduction activities; and (c) partner with health systems to advance risk reduction.

